# Demographic correlates of screen time and objectively measured sedentary time and physical activity among toddlers: a cross-sectional study

**DOI:** 10.1186/s12889-017-4125-y

**Published:** 2017-02-13

**Authors:** Valerie Carson, Nicholas Kuzik

**Affiliations:** Faculty of Physical Education and Recreation, University of Alberta, Edmonton, AB T6G 2H9 Canada

**Keywords:** Toddlers, Physical activity, Sedentary behavior, Correlates, Screen time, Accelerometer

## Abstract

**Background:**

Determining the most important demographic correlates of sedentary behavior and physical activity will help identify the groups of children that are most in need of intervention. Little is known in regards to the demographic correlates of sedentary behavior and physical activity in toddlers (aged 12–35 months), where long-term behavioral patterns may initially be formed. Therefore, the objective of this study was to examine the associations between demographic correlates and specific types of sedentary behavior and physical activity in this age group.

**Methods:**

Findings are based on 149 toddlers (19.0 ± 1.9 months) and their parents (33.7 ± 4.7 years) recruited from immunization clinics in Edmonton, Canada as part of the *Parents’ Role in Establishing healthy Physical activity and Sedentary behavior habits* (PREPS) project. Toddlers’ and parental demographic characteristics and toddlers’ television viewing, video/computer games, and overall screen time were measured via the PREPS parental questionnaire. Toddlers’ objectively measured sedentary time and physical activity (light, moderate to vigorous, and total) were accelerometer-derived using Actigraph wGT3X-BT monitors. Simple and multiple linear regression models were conducted to examine associations.

**Results:**

In the multiple linear regression models, toddlers’ age, toddlers’ sex (female versus male), toddlers’ race/ethnicity (other versus European-Canadian/Caucasian), and household income ($50,001 to $100,000 versus > $100,000) were significantly positively associated, and main type of child care (child care center versus parental care) was significantly negatively associated with screen time. Similar findings were observed with television viewing, except null associations were observed for toddlers’ sex. Toddlers’ race/ethnicity (other versus European-Canadian/Caucasian) was significantly positively associated and main type of child care (child care center, day home, other versus parental care) was significantly negatively associated with video/computer games. Toddlers’ sex (female versus male) was significantly positively associated with sedentary time and significantly negatively associated with moderate- to vigorous-intensity physical activity.

**Conclusions:**

Female toddlers, toddlers from ethnic minority groups, toddlers from families of lower income, and toddlers whose main type of child care is not center-based may be important targets for screen time interventions in toddlers. Apart from sex, demographic correlates may not be important targets for objectively measured sedentary time and physical activity in toddlers.

## Background

Due to the growing evidence and awareness of the importance of regular physical activity and minimal sedentary behavior for optimal growth and development in early years children (<5 years old), countries such as Australia, Canada, and the United Kingdom have guidelines that include recommendations for both these behaviors [1–4]. Most of the research pertaining to guideline adherence in the early years has focused on preschool children [5]. For instance, in both Canada and Australia, ≤15% of preschool children aged 3 to 4 or 3 to 5 years met the primary recommendations in both physical activity (≥180 min/day of total physical activity (TPA)) and sedentary behavior (<1 h/day of screen time) guidelines [6, 7]. The Canadian guidelines also recommend that early years children should progress towards 60 min/day of moderate- to vigorous-intensity physical activity (MVPA) [1]. While 84% of Canadian preschool children from a nationally representative sample participated in ≥180 min/day of objectively measured TPA, only 11% accumulated 60 min/day of MVPA [6]. This suggests that the majority of physical activity that early years children participate in is of light-intensity. Similar patterns have been observed in the limited research conducted in convenience samples of toddlers (aged 12–35 months) that included objective measures of physical activity [8, 9].

The low percentage of early years children meeting both physical activity and sedentary behavior guidelines is a public health concern because behavioral patterns established in early childhood have shown to predict subsequent behavioral patterns later in life [10]. To change behavior, the correlates associated with that behavior need to be identified [11]. Sallis and colleagues suggest that first step in understanding the correlates of behavior is to understand the demographic correlates of behavior so the groups of individuals that are most in need of intervention can be identified [11]. Furthermore, Owen and colleagues have highlighted the importance of behavioral specificity when examining correlates of behavior [12]. For example the correlates of sedentary behavior may differ depending on the type of sedentary behavior (e.g., television versus computer). Similarly, the correlates of physical activity may differ depending on the intensity of activity (e.g., light, MVPA).

In line with guideline adherence work, most of the research on demographic correlates of physical activity and sedentary behavior within the early years, especially for objective measures of these behaviors, has focused on preschool children [13, 14]. The lack of research regarding physical activity and sedentary behavior in toddlers represents an important gap in the current evidence. Toddlers are a particularly important age group for examining physical activity because developmental milestones indicate this is the age when children typically become ambulatory [15]. Furthermore, due to advances in technology (e.g., tablets, smart phones), a large proportion of children begin to engage in regular screen time before the age of 3 years [16, 17]. Therefore, habitual physical activity and sedentary behavior patterns may become established in this age group. Though some evidence exists in older age groups on demographic correlates of specific types of physical activity and sedentary behavior [13, 14], the evidence may not be generalizable to the toddler age group due to their unique developmental period and limited autonomy. Therefore, the objective of this study was to examine the associations between demographic correlates and specific types of sedentary behavior and physical activity among a sample of toddlers aged 12–35 months.

## Methods

### Participants

Participants were parents and their toddlers from the *Parents’ Role in Establishing healthy Physical activity and Sedentary behavior habits* (PREPS) project. Recruitment took place between October, 2014 and December, 2015 at one of four large public health centers in Edmonton, Canada during routine 18-month immunization appointments. The four health centers served areas with diverse socioeconomic and demographic characteristics. To be eligible, toddlers had to be ambulatory and parents had to be English speakers/readers. Eligible families who agreed to participate were asked to complete a consent form and questionnaire during the 15-min wait period required after toddlers’ immunizations. To minimize missing data, research staff checked the questionnaires for completeness at the health centers. A small proportion of families were later contacted via e-mail or phone in regards to missing data. However, participants were informed that they were not required to answer any question they did not want to. At the immunization appointment, families were also given an accelerometer for their toddler to wear for seven consecutive days along with a pre-paid courier envelope so the accelerometer could be mailed back to research staff. Verbal and written accelerometer instructions were provided to families.

A total of 257 out of 491 eligible families (52% participation rate) agreed to participate in the study. Parents who did not agree to participate in the study primarily declined because they were too busy/did not have enough time/were tired (*n* = 74), were not interested in the study (*n* = 64), or thought their toddler would not wear the accelerometer belt (*n* = 60). The remaining participants declined to participate in the study due to travel/illness/moving (*n* = 20) and because a parent was not present (*n* = 16). Ethics approval was obtained from the University of Alberta Human Research Ethics Board and all participating parents provided written informed consent.

### Demographic correlates

#### Toddlers’ characteristics

Parents reported their child’s month and year of birth, sex (‘male’ or ‘female’), race/ethnicity (‘Aboriginal/First Nation’, ‘African-Canadian’, ‘Arabic’, ‘Asian/Pacific Islander’, ‘European-Canadian/Caucasian’, ‘Hispanic/Latino/Latina’, ‘Other’), hours per week spent in care other than the parents (‘day care center’, ‘home daycare’, ‘another adult in your home’, ‘another adult outside your home’, ‘other’), and number of younger (‘0’, ‘1’, ‘2’, ‘3+’) and older siblings (‘0’, ‘1’, ‘2’, ‘3+’). Toddlers’ age in months was calculated using the month and year of birth along with the data collection date. Toddlers’ race/ethnicity was categorized as ‘European-Canadian/Caucasian’ or ‘other’ based on frequency distributions. Toddlers whose parents checked more than one race/ethnicity response were classified as ‘other’. Toddlers’ main type of child care was categorized as ‘parental’, ‘child care center’, ‘day home’, or ‘other’. Parental care was defined as <4 h of non-parental care per week. Child care center and day home were defined as ≥4 h in either center/home day care and <4 h in any other type of care. The remaining toddlers, who received care from a combination of sources or who were cared for primarily by another adult in or outside their home or some other form of care, were categorized as ‘other’. Toddlers’ total number of siblings, including both younger or older were applicable, were categorized as ‘none’, ‘1’, or ‘≥2’. The toddlers’ characteristics questions were adopted from a national survey [18, 19] and have been used in previous research in 0–5 year olds [16, 20].

#### Parental characteristics

Parents reported their birth date, relationship to the toddler (‘mother’, ‘father’, ‘other’), whether they were born in Canada (‘yes’ or ‘no’), marital status (‘married’, ‘living common-law’, ‘widowed’, ‘separated/divorced’, ‘single’, ‘never married’), highest level of education completed (‘no schooling’, ‘elementary’, ‘high school’, ‘community/technical college’, ‘university’, ‘post-graduate’), and gross household income over the past 12 months (‘<$25,000’, ‘$25,000 to $50,000’, ‘$50,001 to $75,000’, ‘$75,001 to $100,000’, ‘>$100,000’, ‘do not know’). Parental age in years was calculated using the birth date along with the data collection date. Parental sex was categorized as ‘female’ and ‘male’ based on whether they responded to being the mother or father of the toddler. No participant selected the ‘other’ response. Parental country of birth was categorized as ‘Canada’ or ‘other’. Parental marital status was categorized as ‘married’ or ‘not married’ based on frequency distributions. Parental education was categorized as ‘high school’, ‘community/technical college’, ‘university’, and ‘post-graduate’ because no included participants had an education below high school. Parental household income was categorized as ‘≤$50.000’, ‘$50,001 to $100,000’, and ‘>$100,000’ based on frequency distributions. For the income variable only, seven participants were excluded because they either did not respond to the question (*n* = 2) or they responded ‘do not know’ (*n* = 5). The parental characteristics questions were adopted from a national survey [18, 19] and have been used in previous research in 0–5 year olds [16, 20].

### Physical activity and sedentary behavior

#### Objectively measured sedentary time and physical activity

Sedentary time and physical activity were objectively measured with waist-worn Actigraph wGT3X-BT accelerometers for 7 consecutive days. Parents were instructed to have their toddler wear the accelerometer continuously for the entire week, except for overnight sleep and during swimming and bathing. Data was collected in 15 s epochs and non-wear time was defined as ≥80 consecutive 15 s intervals of zero counts (equivalent to ≥20 min of consecutive zeros counts). Daytime naps were assumed to be removed with non-wear time. To be included, participants were required to have ≥4 days with ≥ 1440 total 15 s intervals (equivalent to 6 h) of wear time [8, 21]. Sedentary time was defined as 0–24 counts per 15 s, light-intensity physical activity (LPA) as 25–420 counts per 15 s, and MVPA as >420 counts per 15 s [22]. Minutes per day of sedentary time, LPA, and MVPA were calculated by dividing the number of 15 s intervals in each intensity by 4 and then diving by the total number of valid days. Minutes per day of TPA was calculated by adding minutes per day of LPA and MVPA. To adjust for wear time, accelerometer-derived sedentary behavior and physical activity variables were standardized by using the residuals obtained when regressing the variables on wear time [23]. All accelerometer data reduction procedures were performed using SAS version 9.4 (SAS Institute, Cary, NC).

#### Parental reported screen time

Parents reported the average hours and minutes per weekday and weekend day that their toddler watches television, videos, or DVDs on a television, computer, or portable device. Parents also reported the average hours and minutes per weekday and weekend day that their toddler plays video/computer games on devices such as a learning laptop, leapfrog leapster, computer, laptop, tablet, cell phone, the internet, Playstation, or XBOX. Minutes/day of television viewing and video/computer game use were derived by calculating weighted averages for weekday and weekend responses ([weekday*5 + weekend*2]/7). Minutes/day of toddlers’ screen time was calculated by summing the weighted averages of television and video/computer games variables. These questions were modified from a national survey in Canada [6] for a previous study in birth to 5 year olds [16, 20] and have shown good 1-week test re-test reliability in a sub-sample of toddlers from the PREPS project (Carson V, Rhodes RE, Rinaldi C, Rodgers W, Spence JC, Hesketh KD. Reliability of a parental questionnaire for assessing correlates of toddlers’ physical activity and sedentary behavior, submitted).

### Statistical analyses

All statistical analyses were performed using SAS version 9.4 (SAS Institute, Cary, NC). Descriptive statistics were calculated for toddlers’ and parental demographic characteristics. All continuous variables were checked for outliers (≥ ± 3 standard deviations) and sedentary time was truncated below 3 standard deviations for three participants, screen time was truncated below 3 standard deviations for five participants, and MVPA was truncated below 3 standard deviations for one participant. The assumption of normality for regression models was assessed by examining residuals for sedentary behavior and physical activity variables. No variables needed to be transformed. To address the main research objective, simple linear regression models were first conducted between each demographic variable and each sedentary behavior and physical activity variable. Next, multiple linear regression models were run that included all demographic variables that met a cut-off of *p* < 0.10 in the simple linear regression models for each sedentary behavior and physical activity variable. Statistical significance was set at *p* < 0.05.

## Results

Of the eligible 257 participants, 155 participants had complete accelerometer data, as described in the methods section. Four toddlers were excluded because they had a disability that their parent thought could impact their ability to be physically active and two additional toddlers were excluded because they were older than 35 months, leaving a sample of 149 for the final analyses. As described in the methods section, the sample size for all analyses involving parental household income was 142. There were no significant differences for toddlers’ age and sex between those included (*n* = 149) and excluded (*n* = 108) in the final analyses. Descriptive information on toddlers’ and parental characteristics are displayed in Table 1. The average age for toddlers was 19.0 months and for parents was 33.7 years. Just under half (47.6%) of toddlers were female but the majority of parents (87%) were mothers. One quarter (25%) of the sample included parents who were born outside of Canada.

**Table 1:** Toddlers’ and parental demographic characteristics

Demographic variables	(*n* = 149)
Toddlers’ characteristics	
Age (months)	19.0 (±1.9)
Sex	
Male	78 (52.4%)
Female	71 (47.6%)
Race/Ethnicity	
European-Canadian/Caucasian	89 (59.7%)
Other	60 (40.3%)
Main type of child care	
Parental	48 (32.2%)
Child care center	26 (17.5%)
Day home	24 (16.1%)
Other	51 (34.2%)
Number of siblings	
None	61 (40.9%)
1	63 (42.3%)
≥ 2	25 (16.8%)
Parental characteristics	
Age (years)	33.7 (±4.7)
Sex	
Male	19 (12.8%)
Female	130 (87.3%)
Marital status	
Not married	27 (18.1%)
Married	122 (81.9%)
Country of birth	
Canada	111 (74.5%)
Other	38 (25.5%)
Highest level of education	
Post-graduate	35 (23.5%)
University	59 (39.6%)
Community/Technical college	38 (25.5%)
High school (grades 9 –12)	17 (11.4%)
Household income (*n* = 142)	
> $100,000	70 (49.3%)
$50,001 to $100,000	56 (39.4%)
≤ $50,000	16 (11.3%)

Values represent mean (standard deviation) for continuous values, and frequency and percentage for categorical values

The results for the simple linear regression for screen time, television viewing, and video/computer game use are displayed in Table 2. In regards to toddlers’ demographic characteristics, toddlers’ age and race/ethnicity (other versus European-Canadian/Caucasian) were positively associated with screen time, and main type of child care (child care center versus parental care) were significantly negatively associated with screen time. Similar findings were observed for television viewing and video/computer game use. The only exceptions were age was not significantly associated with video/computer games, and compared to parental care, all other main type of child care groups (child care center, day home, other) were significantly negatively associated with video/computer game use. Additionally, the association of sex with screen time and television viewing but not with video/computer games met the *p* < 0.10 cut-off for the multiple regression models. In regards to parental demographic characteristics, country of birth (other versus Canada) and household income (≤$50,000 and $50,001 to $100,000 versus > $100,000 versus) were significantly positively associated with screen time in the simple linear regression models. Similar findings were observed for television viewing and video/computer game use.

**Table 2:** Simple linear regression models for associations of toddlers’ and parental demographic variables with parental-reported screen time, television, and video/computer games

Demographic variables	Screen time (min/day)	Television (min/day)	Video/computer games (min/day)
	β (95% CI)	β (95% CI)	β (95% CI)
Toddlers’ characteristics			
Age (months)	10.1 (3.2, 17.1)**	8.6 (1.8, 15.4)**	2.0 ( −0.7, 4.7)
Sex			
Males	Reference	Reference	Reference
Females	23.9 (−3.5, 51.4)*	22.7 (-4.0, 49.5)*	−1.7 (-9.0, 12.4)
Race/Ethnicity			
European-Canadian/Caucasian	Reference	Reference	Reference
Other	65.7 (39.6, 91.8)**	55.3 (29.3, 81.3)**	19.5 (9.1, 30.0)**
Main type of child care			
Parental	Reference	Reference	Reference
Child care center	−63.1 (-103.2, -23.1)**	−49.2 (-88.7, -9.7)**	−19.6 (-35.1, -4.2)**
Day home	−34.0 (-75.1, 7.1)	−8.3 (-48.9, 32.2)	−21.9 (-37.7, -6.0)**
Other	−22.5 (-55.6, 10.6)	−12.6 (-45.3, 20.0)	−14.6 (-27.4, -1.8)**
Number of siblings			
None	Reference	Reference	Reference
1	6.8 (-23.7, 37.2)	12.5 (-17.1, 42.0)	−4.7 (-16.3, 7.0)
≥ 2	4.5 (-35.7, 44.7)	−6.5 (-45.6, 32.5)	9.4 (-6.0, 24.8)
Parental characteristics			
Age (years)	−1.9 (-4.9, 1.0)	−0.7 (-3.6, 2.2)	−0.7 (-1.9, 0.4)
Sex			
Male	Reference	Reference	Reference
Female	12.0 (-29.5, 53.4)	9.2 (-31.2, 49.6)	7.0 (-9.0, 23.0)
Marital status			
Not married	Reference	Reference	Reference
Married	−17.8 (-53.6, 18.0)	−22.6 (-57.4, 12.2)	−5.9 (-19.7, 8.0)
Country of birth			
Canada	Reference	Reference	Reference
Other	57.0 (26.7, 87.4)**	46.1 (16.1, 76.1)**	21.6 (9.8, 33.3)**
Highest level of education			
Post-graduate	Reference	Reference	Reference
University	17.8 (-18.3, 53.9)	19.7 (-15.4, 54.8)	−3.7 (-17.7, 10.3
Community/Technical college	9.0 (-30.6, 48.6)	7.1 (-31.4, 45.7)	−4.6 (-20.0, 10.8
High school (grades 9-12)	31.2 (-18.8, 81.1)	31.3 (-17.3, 79.9)	−3.8 (-23.3, 15.6
Household income (*n* = 142)			
> $100,000	Reference	Reference	Reference
$50,001 to $100,000	52.4 (23.2, 81.5)**	45.5 (16.8, 74.3)**	13.6 (1.9, 25.3)**
≤ $50,000	58.1 (13.1, 103.1)**	49.2 (4.8, 93.6)**	19.2 (1.1, 37.3)**

*β (95% CI)* unstandardized beta coefficients and 95% confidence intervals, *min/day* minutes per day

**P < 0.05; *P < 0.10

The results for the simple linear regression for objectively measured sedentary time and physical activity are displayed in Table 3. Toddlers’ sex was the only demographic variable significantly associated with any of the objectively measured sedentary time and physical activity variables. More specifically, compared to males (reference group), females engaged in an average 13.9 (95%CI: 0.9, 26.9) more minutes/day of sedentary time and an average 9.3 (95%CI: 3.5, 15.2) less minutes/day of MVPA. The association of sex with TPA and the association of number of siblings with MVPA also met the *p* < 0.10 cut-off for the multiple regression models.

**Table 3:** Simple linear regression models for associations of toddlers’ and parental demographic variables with accelerometer-derived sedentary time and physical activity

Demographic variables	Sedentary time (min/day)^a^	LPA (min/day)^a^	MVPA (min/day)^a^	TPA (min/day)^a^
	β (95% CI)	β (95% CI)	β (95% CI)	β (95% CI)
Toddlers’ Characteristics				
Age (months)	−1.5 (-4.9, 1.9)	1.5 (-0.9, 4.0)	−0.1 (-1.7, 1.5)	1.4 (-2.1, 4.9)
Sex				
Male	Reference	Reference	Reference	Reference
Female	13.9 (0.9, 26.9)**	−3.1 (-12.7, 6.5)	−9.3 (-15.2, -3.5)**	−12.8 (-26.1, 0.5)*
Race/Ethnicity				
European-Canadian/Caucasian	Reference	Reference	Reference	Reference
Other	7.8 (-5.6, 21.2)	−3.5 (-13.3, 6.2)	−4.2 (-10.4, 1.9)	−8.2 (-21.8, 5.5)
Main type of child care				
Parental	Reference	Reference	Reference	Reference
Child care center	−5.8 (−25.5, 13.8)	8.2 (-6.0, 22.5)	0.0 (−8.9, 9.0)	7.5 (−12.5, 27.6)
Day home	5.8 (−14.4, 26.0)	−5.9 (−20.5, 8.7)	2.1 (−7.1, 11.3)	−4.5 (−25.1, 16.0)
Other	6.3 (−9.9, 22.6)	0.3 (−11.4, 12.1)	−5.3 (−12.7, 2.1)	−5.7 (−22.2, 10.8)
Number of Siblings				
None	Reference	Reference	Reference	Reference
1	−8.8 (−23.2, 5.6)	1.1 (−9.4, 11.7)	6.4 (−0.2, 12.9)*	8.0 (−6.7, 22.7)
≥ 2	2.8 (−16.3, 21.8)	−3.2 (−17.2, 10.7)	−0.6 (−9.2, 8.1)	−3.9 (−23.3, 15.6)
Parental Characteristics				
Age (years)	0.8 (−0.7, 2.2)	−0.5 (−1.5, 0.5)	−0.4 (−1.0, 0.3)	0.9 (−2.3, 0.6)
Sex				
Male	Reference	Reference	Reference	Reference
Female	12.9 (−6.7, 32.6)	−10.9 (−25.2, 3.4)	−0.2 (−9.3, 8.9)	−10.8 (−30.9, 9.3)
Marital status				
Not married	Reference	Reference	Reference	Reference
Married	4.5 (−12.6, 21.6)	−0.3 (−12.8, 12.1)	−3.9 (−11.7, 4.0)	−3.9 (−21.3, 13.6)
Country of birth				
Canada	Reference	Reference	Reference	Reference
Other	9.2 (−5.9, 24.3)	−4.3 (−15.3, 6.6)	−4.9 (−11.8, 2.0)	−9.7 (−25.0, 5.7)
Highest level of education				
Post-graduate	Reference	Reference	Reference	Reference
University	7.1 (−10.1, 24.2)	−3.6 (−16.1, 9.0)	−1.6 (−9.5, 6.3)	−6.0 (−23.6, 11.5)
Community/Technical college	13.7 (−5.1, 32.6)	−5.8 (−19.6, 8.0)	−6.0 (−14.6, 2.6)	−12.7 (−32.0, 6.6)
High school (grades 9–12)	1.8 (−22.0, 25.6)	−3.3 (−20.7, 14.1)	0.8 (−10.1, 11.7)	−3.5 (−27.9, 20.8)
Household income (*n* = 142)				
> $100,000	Reference	Reference	Reference	Reference
$50,001 to $100,000	4.8 (−9.5, 19.1)	−2.7 (−13.2, 7.9)	−1.5 (−8.0, 5.0)	−4.7 (−19.3, 9.9)
≤ $50,000	−11.1 (−33.3, 11.0)	6.8 (−9.5, 23.1)	6.5 (−3.5, 16.6)	12.7 (−9.9, 35.2)

*β (95% CI)* unstandardized beta coefficients and 95% confidence intervals, *LPA* light-intensity physical activity, *MVPA* moderate- to vigorous-intensity physical activity, *TPA* total physical activity, *min/day* minutes per day

**P < 0.05; *P < 0.10

^a^Corrected for wear time using the residuals method

The results for the multiple linear regression models for screen time, television viewing, video/computer game use, and MVPA are displayed in Table 4. When adjusting for the other demographic variables in the model, age, sex, race/ethnicity, and main type of child care for toddlers’ demographic characteristics as well as household income for parental demographic characteristics were still significantly associated with screen time in the same direction as the simple linear regression models. More specifically, every additional month of age, screen time was higher by 9.3 (95%CI: 2.8, 15.8) minutes/day. Compared to males (reference group), females engaged in an average 27.1 (95%CI: 2.5, 51.8) more minutes/day of screen time. In comparison to toddlers identified as European-Canadian/Caucasian (reference group), toddlers of all other races/ethnicities engaged in an average 45.7 (95%CI: 17.4, 73.9) more minutes/day of screen time. In comparison to toddlers who received mainly parental care (reference group), toddlers who received care mainly at a child care center engaged in an average 52.5 (95%CI: 16.7, 88.2) less minutes/day of screen time. Finally, in comparison to toddlers from families who had a household income > $100,000 (reference group), toddlers from families who had a household income of $50,001 to $100,000 engaged in an average 39.2 (95%CI: 13.0, 65.5) more minutes/day of screen time. However, significant associations were no longer observed for the lowest income group compared to the highest income group.

**Table 4:** Multiple linear regression models for associations of toddlers’ and parental demographic variables with parental-reported screen time, television, and video/computer games and accelerometer-derived moderate-to vigorous-intensity physical activity

Demographic variables	Screen time (min/day)^b^	Television (min/day)^b^	Video/computer games (min/day)^b^	MVPA (min/day)^a,b^
	β (95% CI)	β (95% CI)	β (95% CI)	β (95% CI)
Toddlers’ characteristics				
Age (months)	9.3 (2.8, 15.8)**	8.0 (1.2, 14.8)**	-	-
Sex				
Male	Reference	Reference	-	Reference
Female	27.1 (2.5, 51.8)**	23.5 (−2.0, 49.1)	-	–8.2 (−14.9, −1.6)**
Race/Ethnicity				
European-Canadian/Caucasian	Reference	Reference	Reference	-
Other	45.7 (17.4, 73.9)**	39.8 (10.4, 69.1)**	14.0 (2.0, 26.0)**	**-**
Main type of child care				
Parental	Reference	Reference	Reference	
Child care center	−52.5 (−88.2, -16.7)**	−39.6 (-76.7, −2.4)**	−17.6 (-32.9, −2.3)**	**-**
Day home	−30.4 (−69.4, 8.6)	−3.9 (−44.4, 36.7)	−18.7 (−35.2, −2.3)**	**-**
Other	−24.1 (−53.5, 5.4)	−13.6 (−44.2, 16.9)	−16.0 (−28.6, −3.4)**	**-**
Number of Siblings				
None	-	-	-	Reference
1	-	-	-	6.6 (−0.6, 13.9)
≥ 2	-	-	-	0.6 (−9.1, 10.3)
Parental characteristics				
Country of Birth				
Canada	Reference	Reference	Reference	-
Other	28.3 (−3.2, 59.9)	22.2 (−10.6, 55.0)	11.1 (−2.3, 24.5)	-
Household income				
> $100,000	Reference	Reference	Reference	-
$50,001 to $100,000	39.2 (13.0, 65.5)**	35.2 (7.9, 62.5)**	8.8 (-2.5, 20.0)	-
≤ $50,000	36.8 (-4.0, 77.6)	31.4 (-11.0, 73.8)	13.8 (-3.7, 31.2)	-

*β (95% CI)* unstandardized beta coefficients and 95% confidence intervals, *min/day* minutes per day, *MVPA* moderate- to vigorous-intensity physical activity

**P < 0.05

^a^Corrected for wear time using the residuals method

^b^Model accounts for 34% of the variance in screen time, 25% of the variance for television viewing, 20% of the variance for video/computer game use, and 7% of the variance for MVPA

The multiple linear regression model findings for television viewing were very similar to screen time, except sex was not significantly associated with television viewing (Table 4). For video/computer games, race/ethnicity and main type of child care were still significantly associated with video/computer game use after adjusting for other demographic variables in the model but household income and parental country of birth were not. For main type of child care, toddlers mainly in a child care center (β = -17.6; 95%CI: −32.9, −2.3), day home (β = -18.7; 95%CI: −35.2, −2.3), and other care (β = -16.0; 95%CI: −28.6, −3.4) all played significantly less minutes/day of video/computer games compared to toddlers mainly in parental care (reference group). Finally, sex was still significantly associated with MVPA, with females engaging in 8.2 (95%CI: 1.6, 14.9) less minutes/day compared to males (reference group). Overall, multiple linear regression models accounted for 34, 25, 20, and 7% of the variance in screen time, television viewing, video/computer game use, and MVPA, respectively. Multiple linear regression models were not conducted for objectively measured sedentary time, LPA, or TPA variables because only one or no demographic variable(s) met the *p* < 0.10 cut-off in the simple linear regression models.

## Discussion

The study fills an important gap in the literature by examining a range of toddlers’ and parental demographic correlates of parental reported television viewing, video/computer game use, and overall screen time as well as objectively measured sedentary time and physical activity in a sample of 149 toddlers. Compared to males, females participated in significantly less MVPA and significantly more sedentary time. Apart from sex, no other demographic variable was independently associated with any of the objectively measured sedentary time and physical activity variables. Females also engaged in significantly more screen time. Similarly, toddlers from ethnic minority groups, toddlers from families of lower income, and older toddlers engaged in significantly more screen time and television viewing. Additionally, toddlers from ethnic minority groups engaged in significantly more video game/computer use. However, in comparison to toddlers mainly cared for by parents, toddlers in the child care center group engaged in significantly less screen time and television viewing and toddlers in the child care center, day home, or other child care groups engaged in significantly less videogame/computer use. Overall, meaningful group differences were observed. For instance, average group differences in overall screen time ranged from 27 to 53 min per day in the multiple linear regression model. Furthermore, this model explained approximately a third of the variance in overall screen time.

Limited studies have objectively measured sedentary time and physical activity in toddlers and examined potential demographic correlates [9, 24, 25]. In line with the present study, Vanderloo and colleagues did not observe any significant associations of ethnicity, child care attendance, family income, and parental education with objectively measured sedentary time, LPA, MVPA, and TPA in a sample of 40 toddlers from London, Canada [9]. Likewise, Johansson and colleagues did not observe significant associations of child care and parental education with objectively measured sedentary time and low and high physical activity variables in a sample of 123 toddlers from Stockholm, Sweden [25]. Similar findings were also observed for parental education and household income with objectively measured sedentary time and MVPA in a sample of 347 toddlers from Rotterdam in the Netherlands [24]. Consistent with the present study, Wijtzes and colleagues found in the Netherlands sample that the percentage of time in sedentary behavior was significantly higher and percentage of time in MVPA was significantly lower in females compared to males [24]. However, sex was not significantly associated with objectively measured sedentary time and physical activity variables in the previous studies from Sweden and Canada [9, 25]. Wijtzes and colleagues also observed that percentage of time in sedentary behavior was lower among older toddlers and toddlers with 2 or more siblings compared to toddlers with no siblings, and the opposite associations were observed for the percentage of time in MVPA [24]. However, age and number of siblings were not significantly associated with any of the objectively measured behaviors in the present study and were not examined in previous studies [9, 25].

Across the limited base of evidence there seems to be few demographic correlates associated with objectively measured sedentary time and physical activity in toddlers. Consistent sex differences in sedentary time and MVPA observed in older children [26, 27] may begin as early as the toddler age group but consistent findings have not been observed across studies. It could be that modifiable correlates are more important for these behaviors than demographic correlates. For instance, Hnatiuk and colleagues reported time spent with babies of a similar age and time spent being physical activity with the mother in infancy was associated with toddlers’ objectively measured TPA [28]. The importance of modifiable correlates needs to be confirmed in future research, given the limited evidence in this age group.

It should also be noted that comparisons of findings across studies that used objective measures of sedentary time and physical activity needs to be made with caution due to different monitors (i.e., Actigraph versus Actical), different monitor placement sites (i.e., hip versus wrist), and different data reduction procedures (i.e., wear time criteria, number of valid days, treatment of daytime naps) used. A number of these factors have shown to impact estimates of sedentary time and physical activity [5, 9]. Additionally, across studies, different procedures or no procedure were used in accounting for accelerometer wear time in the analyses, which may also have impacted findings. While standardizing accelerometer methodologies is a challenge for sedentary behavior and physical activity research across age groups, it is a particular challenge for the toddler age group because of the lack of methodological studies [29]. Therefore, future accelerometer methodological studies in this age group are also needed to strengthen the evidence base around toddlers’ sedentary time and physical activity and the important correlates of these behaviors.

In comparison to objectively measured sedentary time and physical activity, there have been more studies that have examined demographic correlates of screen time in toddlers. Many findings observed in the present study are in line with findings from a recent systematic review, where the evidence on the correlates of screen time in children under 3 years of age was synthesized [30]. For example, age was found to be a consistent positive correlate of screen time, and in terms of race/ethnicity, screen time was consistently found to be higher in non-Caucasian compared to Caucasian participants in the review [30]. Additionally, consistent null associations were observed between number of siblings, and parental education with screen time [30]. It was also reported in the review that unclear findings have been observed for the association of household income with screen time [30]. Significant differences in screen time between household income groups were also not consistently observed for the multiple linear regression models in the present study. However, coefficients did suggest that as household income decreases, screen time increases. Therefore, the sample sizes within each group may have impacted the power to detect significant differences within the present study.

In contrast to the present study, consistent null associations were observed between children’s sex and non-parental care and screen time in the review on correlates of screen time in children under 3 years [30]. However, sex was only significantly associated with screen time in the multiple linear regression models in the present study, suggesting negative confounding of other demographic variables could be present for this relationship. For non-parental care, only one [31] of the five studies included in the review that examined the association between non-parental care and screen time [17, 31–34] compared different types of child care [31]. Similar to the present study, it was found that children in center based care were the least likely to exceed screen time guidelines [31].

Overall the findings of this study and others [30] in regards to the demographic correlates of screen time in toddlers draw attention to some groups of individuals that may be in most need of intervention. More specifically, female toddlers, toddlers from ethnic minority groups, toddlers from families of lower income, and toddlers whose main type of child care is not center-based may be important targets. Additionally, given that age appears to be a consistent positive correlate of screen time in this age group and previous research has shown screen time tracks from early childhood to middle childhood [10], early intervention to promote healthy habits of minimal screen time seems warranted.

It is important to note that the review by Duch and colleagues was not able to examine correlates between infants and toddlers or different types of screen time separately [30]. In fact, only five studies examined correlates of video/computer games in the review. The present study observed that the correlates of television viewing and video/computer game use were not always consistent with overall screen time. Therefore, future research should measure and determine the modifiable and non-modifiable correlates of different types of screen-based sedentary behavior as important intervention targets may not be consistent across all types.

A main strength of this study was the focus on the toddler age group, which fills an important gap in the literature. The inclusion of a wide range of demographic correlates, the recruitment procedures that targeted a socioeconomic and demographically diverse sample, the use of objective measures of sedentary time and physical activity, and the use of a screen time measure that incorporates different types of screen time and newer technology (e.g., smart phones, tablets) are additional study strengths. However, by assuming naps were removed with non-wear time may have introduced some measurement error for the objective measures, especially for sedentary time. Furthermore, the potential bias associated with subjective measures of screen time may have influenced the results. The measure used in the present study has shown good reliability in this age group but the validity of the measure is unknown. Few screen time questionnaires in this age group [30] or older age groups [35] have been validated, and unfortunately no suitable objective measure of screen time for population studies currently exists. Another study limitation is the modest participation rate and the number of participants lost due to incomplete accelerometer measures. However, there were no significant age and sex differences between included and excluded participants. Finally, a limitation inherent among cross-sectional studies is the inability to determine temporality of associations and as a result causality. However, it should be noted that some demographic correlates examined would not have changed (e.g., sex, race/ethnicity); therefore, temporality may be less of a concern for associations observed with these variables.

## Conclusions

Toddlers’ sedentary behavior and physical activity, including the important correlates of these behaviors, is a relatively understudied research area. Findings from the present study suggest that demographic correlates may not be important targets for interventions aiming to decrease objectively measured sedentary time and increase objectively measured physical activity in toddlers. Future research should examine modifiable correlates, such as parental behaviors, parental cognitions, and the home and neighbourhood environment [36]. Conversely, female toddlers, toddlers from ethnic minority groups, toddlers from families of lower income, and toddlers who main type of child care is not center-based may be important targets for screen time interventions. Future research should determine the most important modifiable and non-modifiable correlates of different types of screen-based sedentary behavior.

## Abbreviations

LPA: Light-intensity physical activity
MVPA: Moderate-to vigorous-intensity physical activity
TPA: Total physical activity
